# Caveolin-1 genotypes as predictor for locoregional recurrence and contralateral disease in breast cancer

**DOI:** 10.1007/s10549-023-06919-x

**Published:** 2023-04-05

**Authors:** Christopher Godina, Helga Tryggvadottir, Ana Bosch, Signe Borgquist, Mattias Belting, Karolin Isaksson, Helena Jernström

**Affiliations:** 1 Division of Oncology, Department of Clinical Sciences in Lund, Lund University and Skåne University Hospital, Barngatan 4, 221 85 Lund, Sweden; 2 Department of Hematology, Oncology and Radiation Physics, Skåne University Hospital, Lund and Malmö, Sweden; 3 Department of Oncology, Aarhus University and Aarhus University Hospital, Aarhus, Denmark; 4 Department of Immunology, Genetics and Pathology, Science for Life Laboratory, Uppsala University, Uppsala, Sweden; 5 Division of Surgery, Department of Clinical Sciences in Lund, Lund University and Kristianstad Hospital, Lund and Kristianstad, Sweden

**Keywords:** Caveolin-1, Genotype, Locoregional breast cancer recurrence, Contralateral breast cancer

## Abstract

**Purpose:**

Caveolin-1 (CAV1) has been implicated in breast cancer oncogenesis and metastasis and may be a potential prognosticator, especially for non-distant events. CAV1 functions as a master regulator of membrane transport and cell signaling. Several *CAV1* SNPs have been linked to multiple cancers, but the prognostic impact of *CAV1* SNPs in breast cancer remains unclear. Here, we investigated *CAV1* polymorphisms in relation to clinical outcomes in breast cancer.

**Methods:**

A cohort of 1017 breast cancer patients (inclusion 2002–2012, Sweden) were genotyped using Oncoarray by Ilumina. Patients were followed for up to 15 years. Five out of six *CAV1* SNPs (rs10256914, rs959173, rs3807989, rs3815412, and rs8713) passed quality control and were used for haplotype construction. *CAV1* genotypes and haplotypes in relation to clinical outcomes were assessed with Cox regression and adjusted for potential confounders (age, tumor characteristics, and adjuvant treatments).

**Results:**

Only one SNP was associated with lymph node status, no other SNPs or haplotypes were associated with tumor characteristics. The *CAV1* rs3815412 CC genotype (5.8% of patients) was associated with increased risk of contralateral breast cancer, adjusted hazard ratio (HR_adj_) 4.26 (95% CI 1.86–9.73). Moreover, the TTACA haplotype (13% of patients) conferred an increased risk for locoregional recurrence HR_adj_ 2.24 (95% CI 1.24–4.04). No other genotypes or haplotypes were associated with clinical outcome.

**Conclusion:**

*CAV1* polymorphisms were associated with increased risk for locoregional recurrence and contralateral breast cancer. These findings may identify patients that could derive benefit from more tailored treatment to prevent non-distant events, if confirmed.

**Supplementary Information:**

The online version contains supplementary material available at 10.1007/s10549-023-06919-x.

## Background

Breast cancer remains a clinical challenge. Despite progress in treatment and diagnostics, some patients still relapse [1]. New prognostic and predictive biomarkers are needed to better tailor treatment to the individual patient [1, 2]. While many predictive and prognostic biomarkers exist in breast cancer [3–5], most focus on predicting distant metastasis. No specific biomarker exists for non-distant events, i.e., metachronous contralateral breast cancer or locoregional recurrence [6, 7]. Patients with a metachronous contralateral breast cancer or locoregional recurrence have a higher risk of developing distant metastasis and have worse survival compared to those without [8–10]. By convention, a metachronous contralateral breast cancer is considered a new primary tumor [11]. However, studies have shown that a subset of metachronous contralateral breast cancers represent a metastatic spread of the primary tumor [11, 12]. We previously reported that tumor-specific Caveolin-1 (CAV1) was prognostic for both contralateral breast cancer (CAV1 in malignant cells) and locoregional recurrence (CAV1 in stromal cells) [13]. Furthermore, host factors modulated how CAV1 in malignant and stromal cells affected prognosis [13]. It would, therefore, be of interest to further elucidate the role of CAV1 in breast cancer by studying *CAV1* genotypes.

The *CAV1* gene is located on human chromosome 7(7q31.1) and contains three exons, with the last exon encoding the bulk of the functional domains [14]. CAV1 is primarily located in cholesterol-rich plasma membrane raft domains (caveolae) and serves as a master regulator of cell signaling and transport, including drug internalization [15, 16]. CAV1 is most abundantly expressed in endothelial cells, fibroblasts, and adipocytes [14, 17]. CAV1 and caveolae have been implicated in several vital processes for breast cancer tumorigenesis and invasion, including inflammation, epithelial-mesenchymal transition, hypoxia response, and tumor–stroma interaction [15, 16, 18]. CAV1 has also been linked to radioresistance in various cancers through regulation of tyrosine kinase receptor membrane trafficking and thereby activating DNA repair mechanisms [18]. Moreover, CAV1 plays a crucial role in adipose tissue regulation, which is central to development of metabolic syndrome and obesity [19]. The loss of CAV1 in adipose tissue leads to an inability to store fat properly, leading to lipodystrophy, insulin resistance, hypertriglyceridemia, and metabolic syndrome [19, 20]. CAV1 deficiency in adipose tissue also leads to the recruitment of M2 macrophages [21] that promote tumorigenesis [22]. The role of CAV1 in obesity may be more prominent in women than in men [23]. Therefore, it would be of value to further explore adipose tissue regulators, such as CAV1 in breast cancer, considering the complex relationship between obesity and breast cancer [24, 25]. Specific *CAV1* genotypes are associated with both fat distribution and waist circumference [26]. A meta-analysis showed associations between *CAV1* SNPs and increased risk of breast cancer in Asian and Middle Eastern populations [27], and a similar association between *CAV1* SNPs and gastrointestinal and urinary cancer risk has been reported [28, 29]. However, to our knowledge, there are no studies on the relationship between *CAV1* genotypes and prognosis in breast cancer. Here, we investigated whether *CAV1* genotypes and haplotypes impact prognosis, especially risk for metachronous contralateral breast cancer and locoregional recurrence, in primary breast cancer.

## Materials and methods

### Cohort description

**Fig. 1:**
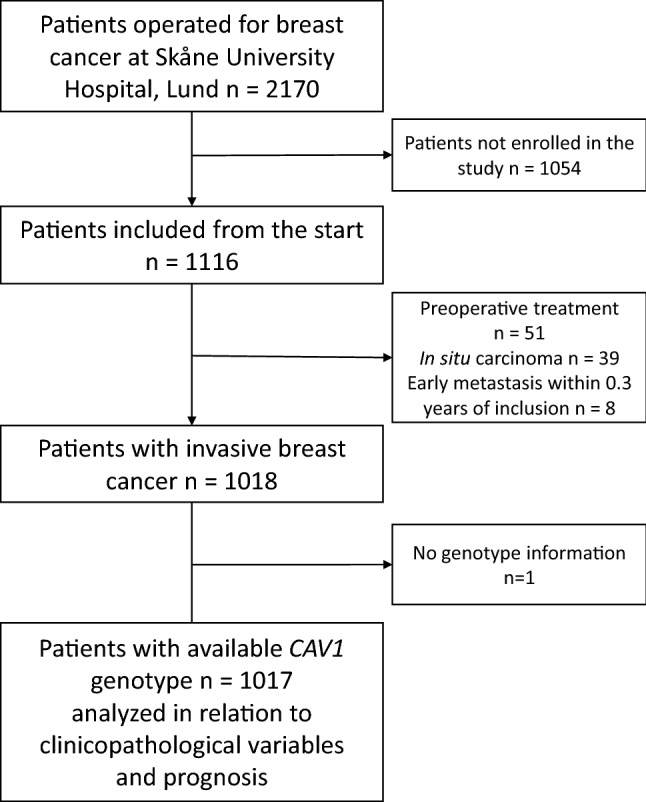
Flowchart of included and excluded patients

BCblood is a population-based breast cancer cohort, consisting of patients with primary breast cancer operated at Skåne University Hospital, Lund. The study was approved by the Lund University Ethics Committee (Dnr 75-02, Dnr 37-08, Dnr 658-09, and amendments). All participants provided written informed consent. Inclusion of patients occurred between diagnosis and surgery. Only patients diagnosed with a first primary breast cancer and had not been diagnosed with cancer 10 years prior were included. At inclusion, a questionnaire regarding lifestyle and reproductive factors was answered, research nurses took anthropometric measurements and collected EDTA plasma for genotyping. Clinical data were obtained from medical records, pathology reports, and registries. After excluding patients with carcinoma in situ, preoperative treatment, and distant metastasis within 0.3 years of inclusion, and no available genotype, 1017 patients remained (inclusion October 2002 to June 2012, (Fig. 1). Last follow-up was June 30, 2019.

Per Swedish clinical routine, the ER and PR positivity cut-offs were > 10% stained nuclei. For patients with missing HER2 status, HER2 status was obtained from dual gene protein staining of HER2 on tissue microarrays, which showed 97.7% agreement with available pathological assessment [30]. Tumor-specific CAV1 staining was obtained and dichotomized, as previously described, into positive/negative for malignant cells and strong/not strong for stromal cells [13, 31]. Anthropometric measurements were dichotomized as in the previous study [13].

### Genotyping

From the leukocyte portion of whole blood, DNA was extracted using DNeasy® Blood and Tissue kit and processed with QiaCube according (Qiagen, Hilden, Germany) according to the manufacturer’s instructions. SNP genotyping was performed by the Centre for Translational Genomics at Lund University using Oncoarray by Illumina [32], specifically designed to evaluate genetic variants for association with the multiple cancers types (including breast). Details on the genotyping calling has been previously described [32]. Standard quality control was performed on all scans. All samples with low call rates (< 1 × 10^–5^), single-nucleotide polymorphisms (SNPs) with minor allele frequency < 1% or call rate < 99% were excluded. For *CAV1* SNPs, genotype intensity cluster plots were examined manually to judge reliability [33]. Five out of six *CAV1* SNPs (rs10256914, rs959173, rs3807989, rs3815412, and rs8713) passed quality control and were in Hardy–Weinberg equilibrium, while the excluded SNP had a minor allele frequency < 1%. The first four SNPs are intronic and rs8713 is a 3’ UTR variant.

### CAV1 haplotype/diplotype construction

**Fig. 2:**
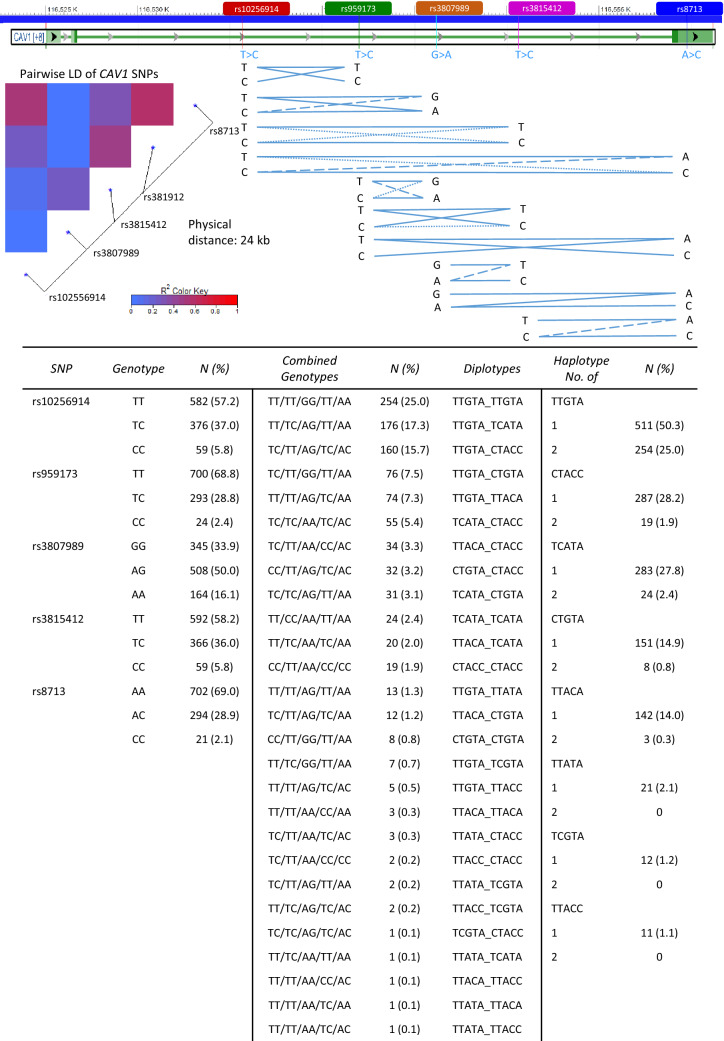
Genomic region of CAV1 along with linkage disequilibrium heatmap and visual illustration of the linkage relationship between *CAV1* SNPs. Continuous lines indicate common combinations while dotted lines indicate less likely combinations. Frequencies of *CAV1* SNPs, combined genotypes, diplotypes, and haplotypes for the 1017 breast cancer patients

Each SNP was cross-tabulated against the other four SNPs and based on the most likely combinations, the haplotypes and diplotypes were constructed. The genotypes for rs10256914 and rs8713 were missing for one patient each and were imputed based on other genotypes (Fig. 2). The haplotypes were compared to a reference European population (1000genome project) from LDlinkR [34] (supplementary table S1). The major allele for all five SNPs were defined according to dbSNP and used as reference for all statistical analyses. Only haplotypes over 10% were analyzed and compared to no copy of each respective haplotype in the analyses. Two haplotypes (CTGTA and TTACA) were dichotomized into any (1+) and none (0) due to low frequency of homozygotes (Fig. 2).

Database searches for proxy and putatively functional variants and expression quantitative trait loci in linkage disequilibrium with the five SNPs were performed using LDLinkR [34] in R (v4.0.2). ‘LDheatmap’ and ‘ggplot2’ were used to generate linkage disequilibrium heatmaps and forest plots, respectively.

### Statistical analyses

The five individual SNPs and derived haplotypes were analyzed in relation to patient and tumor characteristics with chi-square test or linear-by-linear trend test (when appropriate) for categorical variables and Mann Whitney *U*-test or Kruskal–Wallis (when appropriate) for continuous variables.

Endpoints used for survival were locoregional recurrence, contralateral breast cancer, any first breast cancer event, distant metastasis, and death due to any cause. Locoregional recurrence-free interval (LRFI), contralateral breast cancer-free interval (CBCFI), breast cancer-free interval (BCFI), and distant metastasis-free interval (DMFI) were calculated from inclusion until the first event. Patients without any recurrences were censored at the time of the last follow-up before emigration, death, or last follow-up by June 30, 2019. Overall survival (OS) was defined as the time until death or last follow-up by June 30, 2019.

For survival analyses, univariable analyses were conducted with Log-rank tests and Kaplan–Meier curves. For multivariable survival analyses, Cox proportional hazards models were used. Two models were used: model 1 that was adjusted for age and tumor characteristics and model 2 that was further adjusted for adjuvant treatments. Schoenfeld’s residuals were used to test the proportional hazard assumption for the genotypes and haplotypes in model 2. Survival analyses with CBCFI as endpoint were restricted to patients without bilateral tumors. To investigate effect modifications between the *CAV1* genotypes and tumor-specific CAV1 (both in malignant and stromal cells) on clinical outcome, formal two-way interactions analyses were performed in model 2. Further, since radiotherapy is mainly given to prevent locoregional disease, exploratory analysis were also performed stratified by radiotherapy for LRFI to elucidate whether the genotypes were associated with radioresistance [6, 7].

For sensitivity analyses, Fine-Gray subdistribution hazard models for two endpoints (locoregional recurrence and contralateral breast cancer) were fitted and adjusted according to multivariable model 2, to account for death and other types of breast cancer events as a competing risk. Further sensitivity analyses were conducted with additional adjustment for BMI, HER2, and tumor-specific CAV1. To accommodate for missing data for these covariates, multiple imputation by chained equations were used and the pooled results were compared to the complete case results as previously performed [13]. Since the CC genotype was more common among TNBC, an additional analysis of rs3815412 in relation to CBCFI was conducted adjusting for TNBC status.

All statistical analyses were conducted in STATA version 17.0 (StataCorp, College Station, TX, US). A *P* value < 0.05 was considered significant. All *P* values were two tailed. Nominal *P* values are presented without adjustment for multiple testing due to the exploratory nature of this study [35].

## Results

### Patient and tumor characteristics in relation CAV1 genotypes and haplotypes

Database searches revealed that all five *CAV1* SNPs were linked to other genetic variants in *CAV1* regulating its expression in adipocytes, in particular the rs3807989 A-allele and rs3815412 C-allele were linked genotypes associated with lower CAV1 gene expression. None of the five *CAV1* SNPs were associated with patient characteristics. The TTGTA haplotype was associated with age at inclusion (*P* = 0.001), where patients having no haplotype were younger than other patients. No other associations between patient characteristics and haplotypes were found. Moreover, there were no associations between *CAV1* SNPs and haplotypes and tumor characteristics with the exception of an association between rs959173 and nodal status (*P* = 0.032). Tumor-specific strong CAV1 in stromal cells and positive CAV1 in malignant cells were similar across *CAV1* genotypes and haplotypes. Table 1 presents descriptive statistics for all 1017 patients as well as for SNP rs3815412 and the TTACA haplotype, which were related to prognosis.

**Table 1:** Patient and tumor characteristics in relation to rs3815412 genotypes and TTACA haplotype

	All	Missing	rs3815412, *n* = 1017			TTACA haplotype, *n* = 1017	
	Patients		TT	TC	CC	None	Any
	*n* = 1017		*n* = 592 (58.2%)	*n* = 366 (36.0%)	59 (5.8%)	*n* = 872 (85.7%)	*n* = 145 (14.3%)
	Number (%)		Number (%)	Number (%)	Number (%)	Number (%)	Number (%)
	or Median (IQR)		or Median (IQR)	or Median (IQR)	or Median (IQR)	or Median (IQR)	or Median (IQR)
Age at inclusion, years	61.1 (52.1─68.1)	0	60.9 (52.8─67.9)	61.0 (51.3─68.4)	63.0 (50.4─68.4)	60.9 (52.3─68.3)	62.3 (51.6─67.7)
BMI ≥ 25 kg/m^2^	502 (50.8)	28	298 (52.0)	173 (48.5)	31 (52.5)	431 (50.9)	71 (50.0)
Waist circumference ≥ 80 cm	730 (74.6)	38	426 (75.1)	256 (72.3)	48 (82.8)	619 (73.9)	111 (78.7)
Alcohol abstainer, yes	105 (10.4)	3	63 (10.7)	36 (9.9)	6 (10.2)	89 (10.2)	16 (11.0)
Preoperative smoker, yes	206 (20.3)	2	117 (19.8)	76 (20.8)	13 (22.0)	173 (19.9)	33 (22.9)
Oral contraceptives, ever	722 (71.0)	1	416 (70.4)	262 (71.6)	44 (74.6)	621 (73.1)	101 (69.7)
Menopausal hormone therapy, ever	446 (44.0)	3	268 (45.4)	153 (41.9)	25 (42.4)	383 (44.1)	65 (43.5)
Number of children		0					
0 (Nulliparous)	122 (12.0)		74 (12.5)	44 (12.0)	4 (6.8)	104 (11.9)	18 (12.4)
1─2	627 (61.7)		373 (63.0)	216 (59.0)	38 (64.4)	535 (61.4)	92 (63.5)
3+	268 (26.3)		145 (24.5)	106 (29.0)	17 (28.8)	233 (26.7)	35 (24.1)
Screening detected (ages 45─74)	568 (66.2)	159	325 (65.0)	208 (67.5)	35 (70.0)	484 (66.4)	84 (65.6)
Invasive tumor size		0					
pT2/3/4	276 (27.1)	0	151 (25.1)	110 (30.1)	15 (25.4)	232 (26.6)	44 (30.3)
Any axillary lymph node involvement	389 (38.3)	2	227 (38.4)	136 (37.3)	26 (44.1)	330 (37.9)	59 (40.7)
Tumor-specific CAV1							
Strong staining stromal cells	339 (37.1)	103	187 (35.4)	136 (40.7)	16 (30.8)	293 (37.6)	46 (34.3)
Positive staining malignant cells	392 (44.3)	132	219 (42.3)	150 (47.3)	23 (46.0)	335 (44.1)	57 (45.2)
Receptor status							
ER^+^	894 (88.0)	1	522 (88.3)	324 (88.5)	48 (81.4)	768 (88.2)	126 (86.9)
PR^+^	721 (71.0)	1	417 (70.6)	262 (71.6)	42 (71.2)	613 (70.4)	108 (74.5)
HER2 Amplification	109 (11.4)	63	65 (11.7)	37 (10.9)	7 (12.1)	90 (11.0)	19 (14.2)
Triple Negative	74 (7.3)	7	40 (6.8)	26 (7.2)	8 (13.6)	62 (7.2)	12 (8.3)
Main histological type		0					
No special type (formerly ductal)	822 (80.8)		483 (81.6)	292 (79.8)	47 (79.7)	704 (80.7)	118 (81.4)
Lobular	117 (11.5)		64 (10.8)	46 (12.6)	7 (11.9)	102 (11.7)	15 (10.3)
Other or mixed	78 (7.7)		45 (7.6)	28 (7.7)	5 (8.5)	66 (7.6)	12 (8.3)
Histological grade		1					
I	256 (25.2)		146 (24.7)	96 (26.3)	14 (23.7)	222 (25.5)	34 (23.6)
II	504 (49.6)		305 (51.5)	174 (47.7)	25 (42.4)	438 (50.2)	66 (45.8)
III	256 (25.2)		141 (23.8)	95 (26.0)	20 (33.9)	212 (24.3)	44 (30.6)
Ever treatment by last follow-up prior to any event							
Chemotherapy	258 (25.4)	0	148 (25.0)	94 (25.7)	16 (27.1)	219 (25.1)	39 (26.9)
Radiotherapy	644 (63.3)	0	375 (63.3)	224 (61.2)	45 (76.3)	550 (63.1)	94 (64.8
Herceptin	72 (7.1)	0	38 (6.4)	30 (8.2)	4 (6.8)	58 (6.7)	14 (9.7)
ER^+^ tumors							
Tamoxifen	572 (64.0)	0	335 (64.2)	204 (64.2)	29 (60.4)	490 (63.8)	82 (65.1)
Aromatase inhibitor	371 (41.5)	0	222 (42.5)	127 (39.2)	22 (45.8)	315 (41.0)	56 (44.4)

### *CAV1* genotype and haplotype in relation to prognosis

The patients were followed for up to 15 years. Median follow-up for the patients still at risk (*n* = 734) was 9.05 years (interquartile range 7.03–11.1). There were 195 patients with any breast cancer event during follow-up (61 with locoregional recurrence, 48 with contralateral breast cancer, and 122 with distant metastasis). During follow-up, 188 patients died, of which 100 had a prior breast cancer event. The hazards for genotypes and haplotypes were proportional during follow-up.

**Fig. 3:**
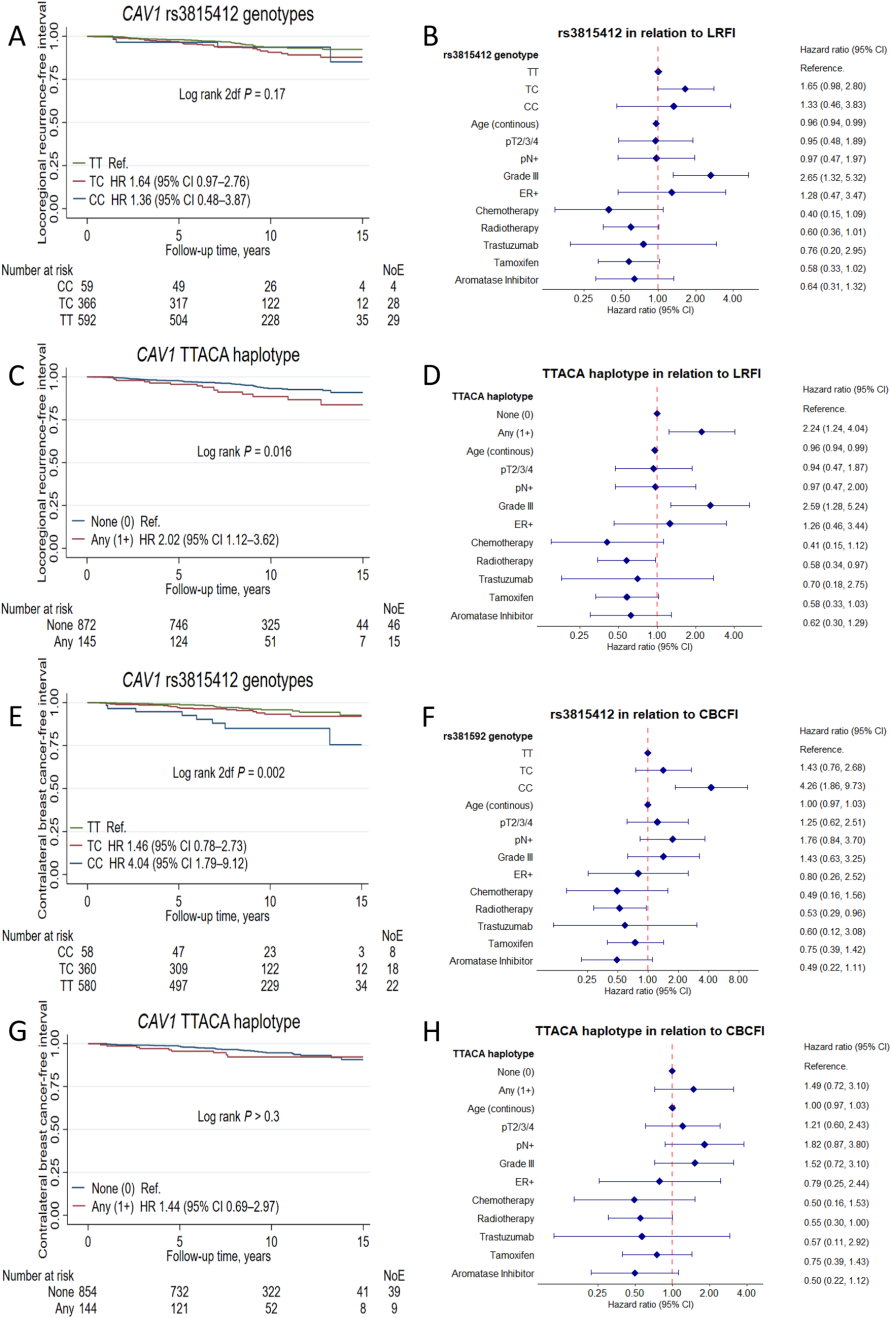
Kaplan–Meier estimates of (**a, c**) locoregional recurrence-free interval with corresponding (**b, d**) forest plots of adjusted hazard ratios (95% confidence intervals), contralateral breast cancer-free interval (**e, g**) with corresponding (**f, h**) forest plots of adjusted hazard ratios (95% confidence intervals) in relation to the *CAV1* rs3815412 genotype and TTACA haplotype in all patients. The number of patients is indicated at each time-point. The study is ongoing; thus, the number of patients decreases with time

The rs3815412 CC genotype was associated with a borderline increased risk of any breast cancer event (Table 2 and supplementary figure S1) that appeared to be driven by an increased risk for contralateral breast cancer, adjusted hazard ratio (HRadj) 4.26 (95% CI 1.86–9.73; Fig. 3). There was no interaction between the rs3815412 SNP and tumor-specific CAV1 in malignant cells on CBCFI. The effect estimates became marginally higher after adjustment for TNBC status. No interaction analysis was performed because there were only three contralateral events in the TNBC subgroup. After further adjustment for BMI, HER2 status, and positive CAV1 cytoplasmic staining in malignant cells, the association remained statistically significant in both the complete case and multiple imputation models (supplementary table S2). Controlling for competing risk (other breast cancer events and death) did not substantially change the effect estimates (supplementary table S3). Furthermore, a weak association between the rs3807989 AA genotype and any breast cancer event was observed (supplementary table S4). However, the AA genotype was in complete linkage with the rs3815412 CC genotype, which appeared to drive the association. No other *CAV1* SNPs were associated with clinical outcome (supplementary table S4).

**Table 2:** Multivariable Cox regression survival analyses of rs3815412 genotypes and the TTACA haplotype in relation to any breast cancer event, distant metastases, locoregional recurrences, contralateral breast cancer, and death due to any cause for the entire follow-up period

Breast cancer event	Total	Events	Crude		Adjusted model 1		Adjusted model 2	
rs3815412	*n*	*n*	HR	(95% CI)	HR	(95% CI)	HR	(95% CI)
TT	592	109	Ref		Ref		Ref	
TC	366	69	0.88	0.65–1.19	1.09	0.80–1.48	1.06	0.78–1.44
CC	59	17	1.68	1.01–2.81	1.58	0.94–2.64	1.55	0.93–2.60

TTACA haplotype	*n*	*n*	HR		HR		HR	
None (0)	872	160	Ref		Ref		Ref	
Any (1 +)	145	135	1.39	0.97–2.01	1.36	0.94–1.96	1.39	0.96–2.01

Distant metastasis								
rs3815412	*n*	*n*	HR	(95% CI)	HR	(95% CI)	HR	(95% CI)
TT	592	74	Ref		Ref		Ref	
TC	366	38	0.87	0.59–1.29	0.86	0.58–1.27	0.84	0.57–1.25
CC	59	10	1.34	0.70–2.61	1.20	0.62–2.35	1.17	0.60–2.28

TTACA haplotype	*n*	*n*	HR		HR		HR	
None (0)	872	172	Ref		Ref		Ref	
Any (1+)	145	23	0.67	0.43–1.03	0.66	0.43–1.03	0.67	0.43–1.04

Locoregional recurrence								
rs3815412	*n*	*n*	HR	(95% CI)	HR	(95% CI)	HR	(95% CI)
TT	592	29	Ref		Ref		Ref	
TC	366	28	1.64	0.97–2.76	1.68	0.99–2.83	1.65	0.98–2.80
CC	59	4	1.36	0.48–3.87	1.37	0.44–3.91	1.33	0.46–3.83

TTACA haplotype	*n*	*n*	HR		HR		HR	
None (0)	872	46	Ref		Ref		Ref	
Any (1+)	145	15	2.02	1.13–3.62	2.15	1.19–3.87	2.24	1.24–4.04

Contralateral breast cancer								
rs3815412	*n*	*n*	HR	(95% CI)	HR	(95% CI)	HR	(95% CI)
TT	592	22	Ref		Ref		Ref	
TC	366	18	1.46	0.78–2.73	1.46	0.78–1.14	1.43	0.76–2.68
CC	59	8	4.04	1.79–9.12	4.05	1.77–9.23	4.26	1.86–9.73

TTACA haplotype	*n*	*n*	HR		HR		HR	
None (0)	872	39	Ref		Ref		Ref	
Any (1+)	145	9	0.67	0.43–1.03	0.66	0.43–1.03	0.67	0.43–1.04

Death								
rs3815412	*n*	*n*	HR	(95% CI)	HR	(95% CI)	HR	(95% CI)
TT	592	118	Ref		Ref		Ref	
TC	366	59	0.87	0.65–1.19	0.85	0.62–1.15	0.85	0.62–1.17
CC	59	11	0.90	0.48–1.65	0.92	0.50–1.73	0.92	0.49–1.73

TTACA haplotype	*n*	*n*	HR		HR		HR	
None (0)	872	163	Ref		Ref		Ref	
Any (1+)	145	25	0.93	0.61–1.41	0.95	0.62–1.45	0.97	0.63–1.48

Adjusted model 1: Age at inclusion, tumor size, nodal status, grade III, and ER status. Missing data for four patients for at least one variable

Adjusted model 2: Model 1 + chemotherapy, radiotherapy, trastuzumab, tamoxifen, and aromatase inhibitors. Missing data for four patients for at least one variable

Among the five common haplotypes, only TTACA was associated with outcome (Table 2 and supplementary table S5). Having at least one copy of the TTACA haplotype conferred borderline increased risk for any breast cancer event HR_adj_ 1.39 (95% CI 0.96–2.01; Table 2 and supplementary figure S2), driven by an increased risk for locoregional recurrence HR_adj_ 2.24 (95% CI 1.24–4.04; Fig. 1 and Table 2). The association was more pronounced in the 366 non-radiotherapy-treated patients HR_adj_ 3.70 (95% CI 1.22–11.21) compared to the 644 radiotherapy-treated patients HR_adj_ 1.80 (95% CI 0.77–4.23) but the effect modification was not significant (*P*_interaction_ = 0.21). There was also no interaction between tumor-specific CAV1 in stromal cells and TTACA haplotype on LRFI. After further adjustment for BMI, HER2 status, and strong CAV1 staining in stromal cells, the association remained statistically significant in both the complete case and multiple imputation models (supplementary table S2). Controlling for competing risks did not substantially change the effect estimates (supplementary table S3).

## Discussion

Both *CAV1* genotypes and haplotypes were associated with risk of metachronous contralateral breast cancer and locoregional recurrence in breast cancer. The rs3815412 CC genotype was associated with a fourfold increased risk for metachronous contralateral breast cancer, and the TTACA haplotype was associated with a twofold increased risk for locoregional recurrence. We previously reported that tumor-specific CAV1 was a predictor for both contralateral breast cancer and locoregional recurrence depending on its localization [13]. The effect of *CAV1* genotypes appeared to be independent of tumor characteristics including CAV1 protein expression. This indicates that host factors and tumor microenvironment may be of importance for predicting metachronous contralateral breast cancer and locoregional recurrence.

The three SNPs rs3807989, rs3815412, and rs8713, not only distinguish the TTACA haplotype from the major haplotype (TTGTA) but also capture the genomic region surrounding the last exon of the *CAV1* gene, which encodes most of the functional domains [14]. None of these five Oncoarray SNPs were in coding regions but may be involved in splicing, transcription and translation of CAV1, regulating the expression of different isoforms.

Especially two of the genotyped SNPs are linked to other SNPs in the *CAV1* gene that regulate CAV1 expression in adipocytes. The genotypes associated with increased risk for non-distant events in our study were associated with lower CAV1 expression in adipocytes. Loss of CAV1 in adipocytes leads to impaired internalization and storage of lipids, lipodystrophy, hypertriglyceridemia, and metabolic syndrome but notably not to increased adiposity [19, 20]. This would correlate to the metabolically obese normal-weight phenotype [36], which constitutes a unique adipose tissue microenvironment similar to obesity induced tumor microenvironment. The metabolically obese normal-weight phenotype is not well captured by BMI [36, 37]. In line with this, we found no association between BMI and *CAV1* genotypes in our cohort.

The effect of the tumor microenvironment caused by the obese normal-weight phenotype on breast cancer is less well understood [36], it is possible that similar mechanisms driving breast cancer progression are at play as in the obese microenvironment [36, 37]. The knockdown of CAV1 leads to increased expression of aromatase in adipocytes [23], increasing the free estrogen in the surrounding tissues promoting breast cancer tumorigenesis [36, 37]. Also, CAV1 deficiency leads to inability to properly stabilize the insulin receptor, rendering the adipocytes unresponsive to insulin [38] and causing inflammation [39]. This tumor promoting inflammation might be mediated by M2 macrophages that promote tumorigenesis [22] and are linked to CAV1 expression in adipocytes [21]. Taken together, this indicates that the obese normal-weight phenotype, which might be captured by the *CAV1* genotype, favors the development of metachronous contralateral breast cancer and locoregional recurrence whereas obesity favors distant recurrences. To summarize, decreased CAV1, which the *CAV1* SNPs were related to, leads to several changes resulting in an unfavorable adipose tissue microenvironment [36] that may promote recurrences in especially in breast tissue, which would explain our findings. The impact of *CAV1* TTACA haplotype on locoregional recurrence risk was less pronounced in radiotherapy-treated patients compared to non-treated patients. The finding merits further investigation to elucidate whether radiotherapy to prevent locoregional recurrences might be especially beneficial for patients with the *CAV1* TTACA haplotype. CAV1 expression in tumors has been linked to radioresistance in several cancers [18]. The relationship between *CAV1* genotypes and radioresistance is still unknown. Further studies are needed.

The strengths of this study includes, a population-based patient cohort considered representative for its catchment area with reliable clinicopathological and anthropometric data [40]. The most common reason for not participating was the lack of available research nurses. Further approximately 5% of patients had an unclear diagnosis at the time of surgery and were therefore not included at the preoperative visit. Previous studies demonstrated that participants of the BCblood cohort were similar to all operated patients with regards to age and hormone receptor status [40, 41]. Additionally, tumor-specific CAV1 data were available [13], enabling a unique dataset with long-term follow-up for analysis. *CAV1* genotyping was done using a SNParray designed to investigate genetic variations in relation to cancer [32]. Nonetheless, it would valuable to investigate in-depth the *CAV1* genomic region to elucidate causal relationships between *CAV1* genotypes, adipocytes, and breast cancer.

Most cases of metachronous contralateral breast cancer are considered to be new primary cancer [11]. This would imply that the rs3815412 CC genotype might be a risk factor for primary breast cancer. To our knowledge, genome-wide association studies did not find associations between CAV1 polymorphisms or the genomic region where it is located and breast cancer risk [42, 43]. However, in case–control and cohort studies, the rs3807987 SNP, which is in linkage with the rs3815412, was associated with breast cancer risk Asian and Middle Eastern populations [27]. Further, several SNPs in multiple genes are more strongly associated with either ER-positive or negative disease [42, 44]. In our cohort, there were very few metachronous contralateral breast cancers in the ER-negative subgroup, making subgroup analyses meaningless. To confirm our findings, large and well-designed studies in various populations are needed.

Metachronous contralateral breast cancer and locoregional recurrence have few established specific prognostic markers, yet impact outcome in breast cancer [8–10]. For locoregional recurrence, prognostic factors related to tumor phenotype have been proposed [45–47]. Specific prognostic factors for metachronous contralateral breast cancer [7] are mostly related to established factors for breast cancer risk. Beyond existing tumor-related prognostic factors, *CAV1* genotypes might offer new prognostic information related to the host.

In conclusion, *CAV1* polymorphisms were shown to be associated with an increased risk for contralateral breast cancer and locoregional recurrence. If confirmed, the findings may identify patients that could derive benefit from more tailored treatment to prevent non-distant breast cancer events.

## Supplementary Information

Below is the link to the electronic supplementary material.

Kaplan-Meier estimates of (A) breast cancer-free interval with corresponding (B) forest plot of adjusted hazard ratios (95% confidence intervals), (C) distant metastasis-free interval with corresponding (D) forest plot of adjusted hazard ratios (95% confidence intervals), (E) overall survival with corresponding (F) forest plot of adjusted hazard ratios (95% confidence intervals) in relation to *CAV1* rs3815412 genotype in all patients. The number of patients is indicated at each time-point. The study is ongoing; thus, the number of patients decreases with time. Supplementary file1 (PDF 356 kb)

Kaplan-Meier estimates of (A) breast cancer-free interval with corresponding (B) forest plot of adjusted hazard ratios (95% confidence intervals), (C) distant metastasis-free interval with corresponding (D) forest plot of adjusted hazard ratios (95% confidence intervals), (E) overall survival with corresponding (F) forest plot of adjusted hazard ratios (95% confidence intervals) in relation to *CAV1* TTACA haplotype in all patients. The number of patients is indicated at each time-point. The study is ongoing; thus, the number of patients decreases with time. Supplementary file2 (PDF 317 kb)

Supplementary file3 (PDF 41 kb)

Supplementary file4 (PDF 74 kb)

Supplementary file5 (PDF 71 kb)

Supplementary file6 (PDF 616 kb)

Supplementary file7 (PDF 606 kb)

## Data Availability

Clinical data are not publicly available due to privacy laws. Questions regarding data can be directed to the corresponding author.

## Abbreviations

BCFI: Breast cancer-free interval
BMI: Body mass index
CAV1: Caveolin-1
CBCFI: Contralateral breast cancer-free interval
DMFI: Distant metastasis-free interval
EDTA: Ethylenediaminetetraacetic acid
ER: Estrogen receptor
HER2: Human epidermal growth factor receptor 2
HR: Hazard ratio
IHC: Immunohistochemistry
LRFI: Locoregional recurrence-free interval
NoE: Number of events
OS: Overall survival
SNP: Single-nucleotide polymorphism
PR: Progesterone receptor
TGFβ: Transforming growth factor-beta
TNBC: Triple-negative breast cancer
