# Supplementary material for: Caveolin-1 genotypes as predictor for locoregional recurrence and contralateral disease in breast cancer
Source: Breast Cancer Res Treat. 2023 Apr 5;199(2):335–47. doi: 10.1007/s10549-023-06919-x (PMC10175335; doi:10.1007/s10549-023-06919-x)
Supplement: Supplementary file 2 — Kaplan-Meier estimates of (A) breast cancer-free interval with corresponding (B) forest plot of adjusted hazard ratios (95% confidence intervals), (C) distant metastasis-free interval with corresponding (D) forest plot of adjusted hazard ratios (95% confidence intervals), (E) overall survival with corresponding (F) forest plot of adjusted hazard ratios (95% confidence intervals) in relation to CAV1 TTACA haplotype in all patients. The number of patients is indicated at each time-point. The study is ongoing; thus, the number of patients decreases with time. Supplementary file2 (PDF 317 kb) [file 10549_2023_6919_MOESM2_ESM.pdf]

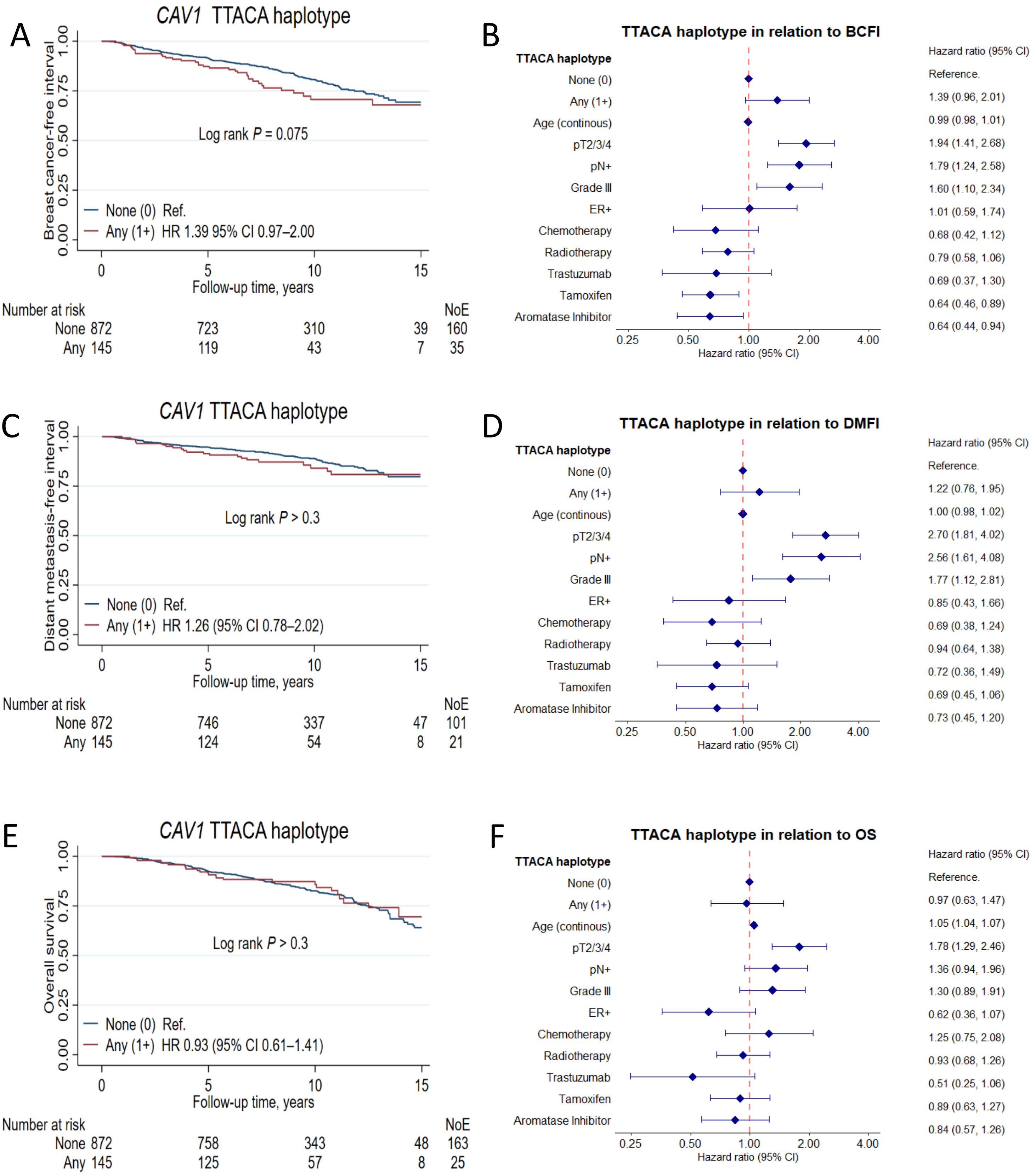

Caveolin-1 genotypes as predictor for locoregional recurrence and contralateral disease in breast cancer
Breast Cancer Research and Treatment
Godina C, Tryggvadottir H, Bosch A, Borgquist S, Belting M, Isaksson K, Jernström H.
H Jernström: Oncology, Department of Clinical Sciences in Lund, Lund University, Sweden Email: helena.jernstrom@med.lu.se
