# Supplementary material for: Caveolin-1 genotypes as predictor for locoregional recurrence and contralateral disease in breast cancer
Source: Breast Cancer Res Treat. 2023 Apr 5;199(2):335–47. doi: 10.1007/s10549-023-06919-x (PMC10175335; doi:10.1007/s10549-023-06919-x)
Supplement: Supplementary file 3 — Supplementary file3 (PDF 41 kb) [file 10549_2023_6919_MOESM3_ESM.pdf]

Supplementary Table 1. Expected haplotypes in the European 1000Genome human population

Haplotypes	rs10256914	rs959173	rs3807989	rs3815412	rs8713	Count	Frequency
No. 1	T	T	G	T	A	515	0.5119
No. 2	C	T	A	C	C	155	0.1541
No. 3	T	C	A	T	A	141	0.1402
No. 4	C	T	G	T	A	86	0.0855
No. 5	T	T	A	C	A	82	0.0815
No. 6	T	T	A	T	A	9	0.0089
No. 7	T	C	G	T	A	6	0.006
No. 8	T	T	A	C	C	6	0.006
No. 9	T	T	G	C	A	3	0.003
No. 10	C	T	A	T	A	2	0.002
No. 11	T	C	A	C	A	1	0.001

Caveolin-1 genotypes as predictor for locoregional recurrence and contralateral disease in breast cancer
Breast Cancer Research and Treatment
Godina C, Tryggvadottir H, Bosch A, Borgquist S, Belting M, Isaksson K, Jernström H.
H Jernström: Oncology, Department of Clinical Sciences in Lund, Lund University, Sweden Email: helena.jernstrom@med.lu.se
