# Supplementary material for: Caveolin-1 genotypes as predictor for locoregional recurrence and contralateral disease in breast cancer
Source: Breast Cancer Res Treat. 2023 Apr 5;199(2):335–47. doi: 10.1007/s10549-023-06919-x (PMC10175335; doi:10.1007/s10549-023-06919-x)
Supplement: Supplementary file 4 — Supplementary file4 (PDF 74 kb) [file 10549_2023_6919_MOESM4_ESM.pdf]

Supplementary Table 2. Multivariable cox models with additional adjustment for BMI, HER2, and tumor-specific CAV1 (stromal and malignant cells) in both complete case and imputation models.

**Locoregional recurrence**								
	**Total**	**Events**	**Model 1**		**Model 2[a]**		**Model 2[b]**	
**TTACA haplotype**	***n***	***n***	**HR**		**HR**		**HR**	
None (0)	872	46	Ref.		Ref.		Ref.	
Any (1+)	145	15	2.02	1.13 – 3.62	2.32	1.12 – 4.43	2.24	1.12 – 3.73
**Contralateral breast cancer**								
**rs3815412**	***n***	***n***	**HR**	**(95% CI)**	**HR**	**(95% CI)**	**HR**	**(95% CI)**
TT	592	22	Ref.		Ref.		Ref.	
TC	366	18	1.46	0.78 – 2.73	1.46	0.86 – 3.53	1.34	0.71 – 2.54
CC	59	8	4.04	1.79 – 9.12	3.83	1.48 – 9.92	4.45	1.94 – 10.20

Model 1: Age, tumor characteristics, treatment

Model 2: model 1 + BMI ≥ 25 kg/m^2, HER2 amplification, and tumor-specific CAV1

a: complete case

b: multiple imputation of all covariates

Caveolin-1 genotypes as predictor for locoregional recurrence and contralateral disease in breast cancer
Breast Cancer Research and Treatment
Godina C, Tryggvadottir H, Bosch A, Borgquist S, Belting M, Isaksson K, Jernström H.
H Jernström: Oncology, Department of Clinical Sciences in Lund, Lund University, Sweden Email: helena.jernstrom@med.lu.se
