# Supplementary material for: Caveolin-1 genotypes as predictor for locoregional recurrence and contralateral disease in breast cancer
Source: Breast Cancer Res Treat. 2023 Apr 5;199(2):335–47. doi: 10.1007/s10549-023-06919-x (PMC10175335; doi:10.1007/s10549-023-06919-x)
Supplement: Supplementary file 5 — Supplementary file5 (PDF 71 kb) [file 10549_2023_6919_MOESM5_ESM.pdf]

Supplementary table 3. Multivariable Cox regression compared to competing risk regression of rs3815412 genotypes and the TTACA haplotype in relation contralateral breast cancer and locoregional recurrences, respectively, for the entire follow-up period.

**Contralateral breast cancer**	**Model 1**		**Model 2**		**Model 3**	
**rs3815412 genotype**	**HR**	**(95% CI)**	**SHR**	**(95% CI)**	**SHR**	**(95% CI)**
TT	Ref.		Ref.		Ref.	
TC	1.43	0.76 – 2.68	1.42	0.74 – 2.70	1.44	0.75 – 2.74
CC	4.26	1.86 – 9.73	3.75	1.52 – 9.26	3.85	1.56 – 9.47
Age (continous)	1.00	0.97 – 1.03	1.01	0.98 – 1.04	1.01	0.97 – 1.04
pT2/3/4	1.25	0.62 – 2.51	1.20	0.58 – 2.48	1.18	0.57 – 2.45
pN+	1.76	0.84 – 3.70	2.01	1.01 – 3.98	1.97	1.01 – 3.84
Grade III	1.43	0.63 – 3.25	1.13	0.46 – 2.77	1.12	0.46 – 2.73
ER^+	0.80	0.26 – 2.52	1.04	0.33 – 3.30	1.11	0.34 – 3.61
Chemotherapy	0.49	0.16 – 1.56	0.51	0.18 – 1.46	0.52	0.18 – 1.49
Radiotherapy	0.53	0.29 – 0.96	0.57	0.30 – 1.07	0.58	0.30 – 1.09
Trastuzumab	0.60	0.12 – 3.08	0.80	0.15 – 4.29	0.85	0.16 – 4.53
Tamoxifen	0.75	0.40 – 1.42	0.74	0.40 – 1.37	0.76	0.42 – 1.40
Aromatase Inibitor	0.49	0.22 – 1.11	0.43	0.19 – 0.95	0.44	0.20 – 0.96
**Locoregional recurrence**	**Model 1**		**Model 2**		**Model 3**	
**TTACA Haplotype**	**HR**	**(95% CI)**	**SHR**	**(95% CI)**	**SHR**	**(95% CI)**
None (0)	Ref.		Ref.		Ref.	
Any (1+)	2.24	1.24 – 4.04	2.01	1.03 – 3.91	2.04	1.05 – 3.96
Age (continous)	0.96	0.94 – 0.99	0.97	0.94 – 1.00	0.97	0.94 – 1.00
pT2/3/4	0.94	0.47 – 1.87	0.74	0.34 – 1.60	0.73	0.34 – 1.58
pN+	0.97	0.47 – 2.00	0.69	0.29 – 1.61	0.68	0.29 – 1.59
Grade III	2.59	1.28 – 5.24	2.06	0.89 – 4.76	2.03	0.88 – 4.67
ER^+	1.26	0.46 – 3.44	1.29	0.38 – 4.36	1.29	0.55 – 3.03
Chemotherapy	0.41	0.15 – 1.12	0.50	0.18 – 1.39	0.50	0.18 – 1.38
Radiotherapy	0.58	0.34 – 0.97	0.50	0.29 – 0.88	0.51	0.29 – 0.90
Trastuzumab	0.70	0.18 – 2.75	0.57	0.11 – 2.95	0.60	0.12 – 3.08
Tamoxifen	0.58	0.33 – 1.03	0.56	0.31 – 1.02	0.57	0.32 – 1.03
Aromatase Inhibitor	0.62	0.30 – 1.29	0.88	0.38 – 2.06	0.89	0.39 – 2.05

Missing data for four patients for at least one variable in the mutlivariable models

Model 1: Cox proportional hazard model

Model 2: Competing risk with other types of events as competing risk to the main outcome

Model 3: Competing risk with other other types of events and death as competing risk to the main outcome

Caveolin-1 genotypes as predictor for locoregional recurrence and contralateral disease in breast cancer
Breast Cancer Research and Treatment
Godina C, Tryggvadottir H, Bosch A, Borgquist S, Belting M, Isaksson K, Jernström H.
H Jernström: Oncology, Department of Clinical Sciences in Lund, Lund University, Sweden Email: helena.jernstrom@med.lu.se
