# Supplementary material for: Caveolin-1 genotypes as predictor for locoregional recurrence and contralateral disease in breast cancer
Source: Breast Cancer Res Treat. 2023 Apr 5;199(2):335–47. doi: 10.1007/s10549-023-06919-x (PMC10175335; doi:10.1007/s10549-023-06919-x)
Supplement: Supplementary file 6 — Supplementary file6 (PDF 616 kb) [file 10549_2023_6919_MOESM6_ESM.pdf]

Supplementary table 4. Multivariable Cox regression survival analyses of *CAV1* SNPs in relation to breast cancer events, distant metastases, and death due to any cause for the entire follow-up period.

**Breast cancer events**								
	**Total**	**Events**	**Crude**		**Adjusted model 1**		**Adjusted model 2**	
**rs1025914**	***n***	***n***	**HR**	**(95% CI)**	**HR**	**(95% CI)**	**HR**	**(95% CI)**
TT	582	118	Ref.		Ref.		Ref.	
TC	376	66	0.88	0.65 – 1.19	0.84	0.62 – 1.14	0.82	0.60 – 1.11
CC	59	11	0.89	0.48 – 1.65	0.93	0.50 – 1.73	0.93	0.50 – 1.73
**rs959173**	***n***	***n***	**HR**		**HR**		**HR**	
TT	700	134	Ref.		Ref.		Ref.	
TC	293	58	1.06	0.78 – 1.44	1.07	0.78 – 1.46	1.12	0.82 – 1.53
CC	24	3	0.65	0.21 – 2.04	0.72	0.23 – 2.25	0.73	0.23 – 2.30
**rs3807989**	***n***	***n***	**HR**		**HR**		**HR**	
GG	345	60	Ref.		Ref.		Ref.	
AG	508	97	1.14	0.83 – 1.58	1.12	0.81 – 1.55	1.12	0.80 – 1.55
AA	164	38	1.48	0.98 – 2.22	1.45	0.97 – 2.19	1.48	0.98 – 2.24
**rs8713**	***n***	***n***	**HR**		**HR**		**HR**	
AA	702	131	Ref.		Ref.		Ref.	
AC	294	60	1.16	0.86 – 1.58	1.13	0.83 – 1.53	1.08	0.79 – 1.47
CC	21	4	1.08	0.40 – 2.92	1.10	0.40 – 2.97	1.05	0.38 – 2.85
**Distant metastasis**								
	**Total**	**Events**	**Crude**		**Adjusted model 1**		**Adjusted model 2**	
**rs1025914**	***n***	***n***	**HR**	**(95% CI)**	**HR**	**(95% CI)**	**HR**	**(95% CI)**
TT	582	76	Ref.		Ref.		Ref.	
TC	376	41	0.86	0.59 – 1.26	0.79	0.54 – 1.16	0.78	0.53 – 1.15
CC	59	5	0.61	0.25 – 1.51	0.71	0.29 – 1.76	0.69	0.28 – 1.73
**rs959173**	***n***	***n***	**HR**		**HR**		**HR**	
TT	700	82	Ref.		Ref.		Ref.	
TC	293	39	1.16	0.79 – 1.69	1.15	0.78 – 1.70	1.19	0.81 – 1.76
CC	24	1	0.36	0.05 – 2.59	0.47	0.07 – 3.38	0.45	0.06 – 3.30
**rs3807989**	***n***	***n***	**HR**		**HR**		**HR**	
GG	345	60	Ref.		Ref.		Ref.	
AG	508	40	1.05	0.70 – 1.57	0.99	0.66 – 1.48	0.98	0.65 – 1.49
AA	164	22	1.22	0.73 – 2.07	1.20	0.71 – 2.03	1.20	0.71 – 2.04
**rs8713**	***n***	***n***	**HR**		**HR**		**HR**	
AA	702	86	Ref.		Ref.		Ref.	
AC	294	35	1.03	0.69 – 1.52	0.95	0.64 – 1.41	0.93	0.63 – 1.38
CC	21	1	0.37	0.05 – 2.65	0.41	0.06 – 2.98	0.39	0.05 – 2.81
**Death**								
	**Total**	**Events**	**Crude**		**Adjusted model 1**		**Adjusted model 2**	
**rs1025914**	***n***	***n***	**HR**	**(95% CI)**	**HR**	**(95% CI)**	**HR**	**(95% CI)**
TT	582	114	Ref.		Ref.		Ref.	
TC	376	68	0.94	0.70 – 1.28	0.90	0.66 – 1.22	0.89	0.65 – 1.21
CC	59	6	0.48	0.21 – 1.09	0.49	0.22 – 1.12	0.49	0.22 – 1.13
**rs959173**	***n***	***n***	**HR**		**HR**		**HR**	
TT	700	125	Ref.		Ref.		Ref.	
TC	293	60	1.16	0.85 – 1.57	1.16	0.85 – 1.59	1.17	0.85 – 1.60
CC	24	3	0.77	0.25 – 2.44	0.85	0.27 – 2.68	0.90	0.28 – 2.87
**rs3807989**	***n***	***n***	**HR**		**HR**		**HR**	
GG	345	64	Ref.		Ref.		Ref.	
AG	508	97	1.07	0.78 – 1.46	0.99	0.72 – 1.37	1.00	0.73 – 1.39
AA	164	27	0.95	0.61 – 1.50	1.00	0.64 – 1.58	1.02	0.65 – 1.61
**rs8713**	***n***	***n***	**HR**		**HR**		**HR**	
AA	702	133	Ref.		Ref.		Ref.	
AC	294	54	1.03	0.75 – 1.41	0.98	0.71 – 1.36	0.97	0.71 – 1.35
CC	21	1	0.24	0.03 – 1.74	0.27	0.04 – 1.97	0.28	0.04 – 1.98

Adjusted model 1: Age at inclusion, tumor size, nodal status, grade III, and ER status. Missing data for four patients for at least one variable.

Adjusted model 2: Model 1+ chemotherapy, radiotherapy, trastuzumab, tamoxifen, and aromatase inhibitors. Missing data for four patients for at least one variable.

Caveolin-1 genotypes as predictor for locoregional recurrence and contralateral disease in breast cancer
Breast Cancer Research and Treatment
Godina C, Tryggvadottir H, Bosch A, Borgquist S, Belting M, Isaksson K, Jernström H.
H Jernström: Oncology, Department of Clinical Sciences in Lund, Lund University, Sweden Email: helena.jernstrom@med.lu.se
