# Supplementary material for: Caveolin-1 genotypes as predictor for locoregional recurrence and contralateral disease in breast cancer
Source: Breast Cancer Res Treat. 2023 Apr 5;199(2):335–47. doi: 10.1007/s10549-023-06919-x (PMC10175335; doi:10.1007/s10549-023-06919-x)
Supplement: Supplementary file 7 — Supplementary file7 (PDF 606 kb) [file 10549_2023_6919_MOESM7_ESM.pdf]

Supplementary Table 5. Multivariable Cox regression survival analyses of *CAV1* haplotypes in relation to breast cancer events, distant metastases, and death due to any cause for the entire follow-up period.

**Breast cancer events**								
Number of Haplotypes	**Total**	**Events**	**Crude**		**Adjusted model 1**		**Adjusted model 2**	
**TTGTA**	***n***	***n***	**HR**	**(95% CI)**	**HR**	**(95% CI)**	**HR**	**(95% CI)**
0	252	51	Ref.		Ref.		Ref.	
1	511	96	0.90	0.64 – 1.27	0.89	0.64 – 1.26	0.90	0.64 – 1.26
2	254	48	0.89	0.60 – 1.32	0.91	0.61 – 1.35	0.89	0.59 – 1.32
**TCATA**	***n***	***n***	**HR**		**HR**		**HR**	
0	710	135	Ref.		Ref.		Ref.	
1	283	57	1.08	0.79 – 1.47	1.09	0.80 – 1.49	1.13	0.83 – 1.56
2	24	3	0.65	0.21 – 2.05	0.72	0.23 – 2.26	0.73	0.23 – 2.30
**CTACC**	***n***	***n***	**HR**		**HR**		**HR**	
0	711	135	Ref.		Ref.		Ref.	
1	287	56	1.08	0.79 – 1.48	1.06	0.77 – 1.44	1.03	0.75 – 1.41
2	19	4	1.14	0.42 – 3.07	1.22	0.45 – 3.31	1.15	0.42 – 3.11
**CTGTA**	***n***	***n***	**HR**		**HR**		**HR**	
None (0)	858	172	Ref.		Ref.		Ref.	
Any (1+)	159	23	0.67	0.43 – 1.03	0.66	0.43 – 1.03	0.67	0.43 – 1.04
**Distant metastasis**								
Number of Haplotypes	**Total**	**Events**	**Crude**		**Adjusted model 1**		**Adjusted model 2**	
**TTGTA**	***n***	***n***	**HR**	**(95% CI)**	**HR**	**(95% CI)**	**HR**	**(95% CI)**
0	252	32	Ref.		Ref.		Ref.	
1	511	58	0.88	0.57 – 1.35	0.84	0.54 – 1.30	0.85	0.55 – 1.32
2	254	32	0.96	0.58 – 1.58	0.99	0.60 – 1.64	0.99	0.60 – 1.63
**TCATA**	***n***	***n***	**HR**		**HR**		**HR**	
0	710	83	Ref.		Ref.		Ref.	
1	283	38	1.16	0.79 – 1.70	1.15	0.78 – 1.70	1.19	0.80 – 1.76
2	24	1	0.36	0.05 – 2.59	0.47	0.06 – 3.37	0.45	0.06 – 3.28
**CTACC**	***n***	***n***	**HR**		**HR**		**HR**	
0	711	89	Ref.		Ref.		Ref.	
1	287	32	0.94	0.63 – 1.41	0.89	0.59 – 1.34	0.88	0.59 – 1.32
2	19	1	0.38	0.05 – 2.74	0.47	0.07 – 3.38	0.43	0.43 – 3.10
**CTGTA**	***n***	***n***	**HR**		**HR**		**HR**	
None (0)	858	106	Ref.		Ref.		Ref.	0.50 – 1.38
Any (1+)	159	16	0.77	0.46 – 1.31	0.78	0.46 – 1.33	0.79	0.46 – 1.33
**Death**								
Number of Haplotypes	**Total**	**Events**	**Crude**		**Adjusted model 1**		**Adjusted model 2**	
**TTGTA**	***n***	***n***	**HR**	**(95% CI)**	**HR**	**(95% CI)**	**HR**	**(95% CI)**
0	252	40	Ref.		Ref.		Ref.	
1	511	98	1.17	0.81 – 1.69	1.11	0.77 – 1.61	1.13	0.79 – 1.64
2	254	50	1.21	0.79 – 1.83	1.22	0.80 – 1.86	1.21	0.80 – 1.85
**TCATA**	***n***	***n***	**HR**		**HR**		**HR**	
0	710	126	Ref.		Ref.		Ref.	
1	283	59	1.17	0.86 – 1.61	1.18	0.86 – 1.61	1.18	0.86 – 1.61
2	24	3	0.78	0.25 – 2.45	0.85	0.27 – 2.68	0.91	0.29 – 2.87
**CTACC**	***n***	***n***	**HR**		**HR**		**HR**	
0	711	135	Ref.		Ref.		Ref.	
1	287	52	1.01	0.73 – 1.39	0.99	0.71 – 1.36	0.99	0.71 – 1.37
2	19	1	0.25	0.04 – 1.81	0.29	0.04 – 2.05	0.29	0.04 – 2.09
**CTGTA**	***n***	***n***	**HR**		**HR**		**HR**	
None (0)	858	163	Ref.		Ref.		Ref.	
ANy (1+)	159	25	0.76	0.50 – 1.17	0.71	0.46 – 1.09	0.70	0.46 – 1.08

Adjusted model 1: Age at inclusion, tumor size, nodal status, grade III, and ER status. Missing data for four patients for at least one variable.

Adjusted model 2: Model 1+ chemotherapy, radiotherapy, trastuzumab, tamoxifen, and aromatase inhibitors. Missing data for four patients for at least one variable.

Caveolin-1 genotypes as predictor for locoregional recurrence and contralateral disease in breast cancer
Breast Cancer Research and Treatment
Godina C, Tryggvadottir H, Bosch A, Borgquist S, Belting M, Isaksson K, Jernström H.
H Jernström: Oncology, Department of Clinical Sciences in Lund, Lund University, Sweden Email: helena.jernstrom@med.lu.se
